# Living on Excitement

**Published:** 1860-01

**Authors:** 


### LIVING ON EXCITEMENT.

He lives the longest who eats plain, substantial food, and drinks pure water, other things being equal. But many prefer highly-seasoned and mixed dishes and stimulating drinks. All such persons die before their time, usually from inanition or wasting disease of the bowels. As certainly will the mind suffer declining vigor and efficiency, its stimulants being novel-reading and a morbid thirst for new things.

In the moral or spiritual world the general principle holds true; hence, those who feed on the "pure milk of the word," who travel in the "old paths," are the surest to grow in the exercise and practice of principles, stern, high, and life-giving. What highly-seasoned food and stimulating drinks are to the body, what novel-reading is to the mind, sensation preaching is to the heart; and yet after "these three," the great world, the masses run with eager pace. It is suggested that the clergy should do all in their power to put down the last practice, by not allowing it to be heralded in the papers when, or where, or on what subjects they are to preach. That is the best "society" which always attends its own meetings when its own doors are opened, and which seldom attends any others. Gadding about creates a pernicious excitement, it unsettles and dissatisfies. Let every man attend religious services as a matter of course, the matter of worship, of prayer and praise and meditation being the absorbing objects; all other things being considered as unimportant incidentals. Let no man inquire whether "Paul or Apollos or Cephas" is to preach, and let him take it for granted that the great theme shall be, "The Lamb of God which taketh away the sin of the world."

How wide is the departure from these wholesome ways, may be estimated from the fact, that in a secular daily newspaper for Saturday, there are over fifty "notices" under the "religious" head, of places, themes, and preachers, but not one of them announces a discourse on the subject of "Christ, and him crucified." The whole of them ranging, up or down, from John Brown to the devil. Let no one imagine from this expression, that on the great subject of black and white we are "on the fence." This would do us injustice. We are very decided in our opinions. The first third of a century of our life we passed in the very midst of the "peculiar institution," and another third we have reason to believe will be passed out of it, hence we have had unusual facilities of personal observation, and therefore from a broad and liberal view of the whole question, we most unhesitatingly declare that we are on the other side; and lest this should not be explicit enough for some, we will further add that we are on the right side, so that our friends North and South may hereafter know where to find us, that is, not on the fence, but on the other side of it, the right side.

